# Temperature-induced changes in treatment efficiency and microbial structure of aerobic granules treating landfill leachate

**DOI:** 10.1007/s11274-016-2046-z

**Published:** 2016-04-27

**Authors:** Dorian Mieczkowski, Agnieszka Cydzik-Kwiatkowska, Paulina Rusanowska, Piotr Świątczak

**Affiliations:** Department of Environmental Biotechnology, University of Warmia and Mazury in Olsztyn, Słoneczna 45 G, 10-709 Olsztyn, Poland

**Keywords:** Aerobic granules, Denitrifiers, FISH, Landfill leachate, Temperature

## Abstract

This paper investigates the effect of temperature on nitrogen and carbon removal by aerobic granules from landfill leachate with a high ammonium concentration and low concentration of biodegradable organics. The study was conducted in three stages; firstly the operating temperature of the batch reactor with aerobic granules was maintained at 29 °C, then at 25 °C, and finally at 20 °C. It was found that a gradual decrease in operational temperature allowed the nitrogen-converting community in the granules to acclimate, ensuring efficient nitrification even at ambient temperature (20 °C). Ammonium was fully removed from leachate regardless of the temperature, but higher operational temperatures resulted in higher ammonium removal rates [up to 44.2 mg/(L h) at 29 °C]. Lowering the operational temperature from 29 to 20 °C decreased nitrite accumulation in the GSBR cycle. The highest efficiency of total nitrogen removal was achieved at 25 °C (36.8 ± 10.9 %). The COD removal efficiency did not exceed 50 %. Granules constituted 77, 80 and 83 % of the biomass at 29, 25 and 20 °C, respectively. Ammonium was oxidized by both aerobic and anaerobic ammonium-oxidizing bacteria. *Accumulibacter* sp., *Thauera* sp., cultured *Tetrasphaera* PAO and *Azoarcus*–*Thauera* cluster occurred in granules independent of the temperature. Lower temperatures favored the occurrence of denitrifiers of *Zooglealineage* (not *Z. resiniphila*), bacteria related to *Comamonadaceae*, *Curvibacter* sp., *Azoarcus* cluster, *Rhodobacter* sp., *Roseobacter* sp. and *Acidovorax* spp. At lower temperatures, the increased abundance of denitrifiers compensated for the lowered enzymatic activity of the biomass and ensured that nitrogen removal at 20 °C was similar to that at 25 °C and significantly higher than removal at 29 °C.

## Introduction

Landfilling of waste generates a number of problems, one of which is the creation of landfill leachate. Leachate from the landfill comes mainly from rainwater infiltration, moisture contained within the waste, and the changes taking place in the pile. Several methods are used in landfill operation to deal with leachate. These include sealing the site with clay, using membrane systems to prevent leakage of leachate into the soil, and leachate drainage systems or drainage ditches. Landfill leachate contains organic substances, nitrogen compounds, phosphorus, inorganic substances and heavy metal ions. The composition of landfill leachate is variable, depending on the location, construction of the landfill, its age and water balance, and the type of waste that is deposited there. This makes it difficult to develop a universal method of disposal.

Leachate from an intermediate age landfill is characterized by high COD (2000–20,000 mg/L), low BOD_5_ (500–1000 mg/L) and fairly high NH_4_^+^-N (1000–4000 mg/L) (Wang et al. 2014). The bioavailability of leachate, characterized by the BOD_5_/COD ratio, declines with age of the landfill due to a decreasing share of low molecular weight organic compounds in the leachate (Kulikowska 2009). Landfilling lead to the formation of macromolecular compounds such as humic acids, able to provide up to 60 % of dissolved organic carbon (Wu et al. 2004). Therefore, biological methods are generally recommended for young landfill leachate with a high BOD_5_/COD ratio.

Due to high ammonium concentration and low biodegradable organic matter, removal of nitrogen from landfill leachate by conventional nitrification and denitrification processes using activated sludge is difficult. In wastewater treatment aerobic granular sludge is an emerging type of microbial structure. The outer layers of granules are settled with aerobic microorganisms, while their interior is mostly settled with facultative organisms (Gao et al. 2011). The cooperation of different groups of microorganisms in granules enables efficient pollutant removal. Microorganisms embedded in matrix of extracellular polymers that form granule structure are protected from environmental disturbances. However, even in aerobic granular sludge reactors high ammonium concentration can cause problems with ammonium removal. Aerobic granular sludge has been proved to be a recyclable adsorbent for ammonium from high-strength wastewaters. At levels of <300 mg NH_4_^+^-N/L ammonium is adsorbed by ion exchange mechanism, but when ammonium reaches level of >1000 mg N-NH_4_^+^/L the process is dominated by multilayer, physical adsorption mechanism (Yu et al. 2014).

So far, many laboratory scale studies have been performed to investigate aerobic granular sludge formation and efficiency of pollutant removal at room temperature where heating of the reactor, as an expensive additional cost, was abandoned (Mosquera-Corral et al. 2005; Kishida et al. 2009). Some studies point out that elevated temperature affects positively efficiency of wastewater treatment by aerobic granules. However, most of these studies were conducted with domestic wastewater or with wastewater with moderate of high COD/N ratio (Bassin et al. 2011; Halim et al. 2015). Song et al. (2009) have proved that 30 °C was optimal operational temperatures (out of 25, 30, and 35 °C) because of better settling abilities, bioactivity and compact structure of granules than in other temperatures. Their results indicated that the majority of dominant microbes belonged to *Actinobacteria* and *Proteobacteria*, and they suggest that different bacteria might play important roles at different temperatures. Temperature influences settling characteristics of biomass, for the same granules a twofold slower settling velocity was noted when the water temperature decreased from 40 to 5 °C (Winkler et al. 2012). de Kreuk et al. (2005) have observed that lowering operational temperature from 20 to 8 °C decreased overall ammonium consumption for about 30 %; however, overall nitrification efficiency was only slightly influenced, since decreased microbial activity in the outer layers of granules resulted in a better penetration depth of oxygen. Authors point out that at the temperature of 8–15 °C abnormal and irregular structure of the granules due to filamentous bacteria occurrence causes biomass elution and unsatisfactory denitrification and decontamination parameters. Temperature determinates the phosphate and glycogen accumulating organisms (PAO–GAO) competition in granules. Analysis of different depths of the sludge bed using fluorescent in situ hybridization (FISH) revealed that bottom granules contained considerably more *Accumulibacter* (PAOs) compared to top granules that were dominated by *Competibacter* (GAOs). Removal of GAO dominated sludge resulted in 100 % P-removal efficiencies at 30 °C (Winkler et al. 2011).

In the present work, leachate from a windrow in municipal landfill was biologically treated with aerobic granular sludge. The aim of this study was to assess how the efficiency and kinetics of biological treatment of landfill leachate change and microbial community in aerobic granules adapt to lowering operational temperature in the reactor.

## Materials and methods

### Experimental set-up

The study was conducted with aerobic granules in a cylindrical laboratory scale reactor (GSBR) with a total capacity of 9 L that was aerated by fine bubble diffusers with an aeration rate of 5 L/min. The ratio of reactor height to diameter was 2.2. For sewage supply and disposal, peristaltic pumps were used. The process was fully automated and the temperature was maintained using a cylindrical immersion heater (length 5 cm, diameter 1 cm) attached to the inner reactor wall. The presence of the heater did not disturb efficient mixing of the GSBR. The reactor configuration was as follows: an 8-h cycle, a working volume of 7 L, and a 43 %/cycle of volumetric exchange rate. The reactor cycle consisted of the following phases: 3 min of sedimentation, 10 min of discharge of effluent, 40 min of anoxic filling and 427 min of aeration. The aerobic granular sludge used to inoculate the reactor came from an earlier experiment (Cydzik-Kwiatkowska et al. 2013). The average diameter of biomass in the reactor with inoculum was about 1 mm and the reactor was operated at about 6 g TSS/L (VSS = 0.65 TSS), organics loading rate of 3.35 kg COD/m^3^ day, nitrogen loading rate of 1.1 kg TKN/m^3^ day and hydraulic retention time of 12.8 h.

As the substrate, landfill leachate from the B2 windrow of the “Ekodolina” landfill in Łężyce (Poland) was used. The windrow is uncovered and has been in constant operation since 2003. It has a full sealing system and is drained and degassed. In April of 2010 a composting building was opened, which reduced the biodegradable fraction (20–60 mm) deposits in the landfill. Leachate were diluted (1:1 with tap water) to ensure efficient removal of ammonium nitrogen (Wei et al. 2012). Pollution indicators measured in the diluted leachate were as follows: 937.1 ± 128.2 mg COD/L, 589.7 mg BOD_5_/L, 785 mg BOD_20_/L, 440.8 ± 28.4 mg TKN/L, 303.8 ± 23.1 N-NH_4_^+^/L, alkalinity 20 meq/L. To provide an additional source of inorganic carbon for nitrification, based on previous experiments, 15 mL/L of sodium carbonate and bicarbonate at concentrations of 60.8 and 81.1 mg/L, respectively, were added to diluted leachate. The leachate had an extremely intensive odor. The study was conducted in three stages. In the first, the operating temperature of the reactor was maintained at 29 °C, in the second, it was lowered to 25 °C, and in the third, it was further lowered to the ambient temperature of 20 °C. Each stage was finished when the values of pollutant concentration in the effluent did not vary for more than 10 % within at least 2 weeks. Therefore the first, second and third stage lasted for 80, 140 and 110 days, respectively. Wastewater and biomass in the reactors were analyzed in accordance with APHA (1998). Ammonium was measured by Nesslerization method or distillation method, nitrites and nitrates by colorimetric methods and COD by dichromate method. BOD of wastewater was measured using OxiTop Control system (WTW GmbH). The biomass concentration in the reactor was not regulated. Dissolved oxygen (DO) concentration was measured using a ProODO (YSI Environmental).

### Fluorescence in situ hybridization (FISH)

Biomass samples for molecular analysis were taken in duplicate at the end of each stage and fixed with 4 % paraformaldehyde. Subsequently samples were washed in water and preserved in ethanol–water until its use. Before cutting granules were washed three times in phosphate buffer saline (PBS). The granules were embedded in Cryomatrix Shandon (Thermo Scientific) tissue freezing medium and frozen for 1 h at −20 °C. These frozen granules were sectioned into 20 μm thin slices using a Cryotome FSE (Thermo Scientific) and kept on gelatin-coated microscope slides. FISH was performed according to Nielsen et al. (2009). For the hybridization procedure, the slides were dried in ethanol (50, 80, 100 %). The formamide concentration for the hybridization (2 h, 46 °C) and NaCl concentration for the washing (15 min, 48 °C) are listed in Table 1. The probes were labeled with Cy3 or FLUOS fluorochromes. Samples were analyzed with a Nikon Eclipse epifluorescence microscope (Nikon) equipped with filters suited for Cy3 as well as FLUOS. Vectashield (Vector Laboratories) was used to mount the samples prior to visualization. The FISH-defined populations (Cy3- or FLUOS-labeled) were quantified by image analysis using the software ImageJ (http://rsb.info.nih.gov/ij/) and calculated as biovolume by the percentage of the total area fluorescing with the EUBmix, based on examination of at least 24 fields of view for each probe. The biovolume of all bacteria in the sludge was determined by the ratio between cells targeted by the probe mixture EUBmix and by DAPI staining (4′6-diamidino-2-phenylindole).

**Table 1:** 16S rRNA oligonucleotide probes used for the identification of bacteria

Probe name	Target	Sequence 5′–3′	Formamide (%)/NaCl (mM)	Reference
EUBmix	All *Bacteria*	EUB338 (GCTGCCTCCCGTAGGAGT), EUB338-II (GCAGCCACCCGTAGGTGT), EUB338-III (GCTGCCACCCGTAGGTGT)	0–55/900–20	Amann et al. (1990) and Daims et al. (1999)
nso190	Betaproteobacterial ammonium-oxidizing bacteria	CGATCCCCTGCTTTTCTCC	55/20	Mobarry et al. (1996)
Amx368	All ANAMMOX bacteria	CCTTTCGGGCATTGCGAA	15/318	Schmid et al. (2003)
PAR651	*Paracoccus* sp.	ACCTCTCTCGAA	40/56	Neef et al. (1996)
G_Rb	*Rhodobacter* sp., *Roseobacter* sp.	CTCCAGGTCAGTATCGAGCCAGTGAG	30/112	Giuliano et al. (1999)
Curvi997	*Curvibacter* sp.	CTCTGGTAACTTCCGTAC	35/80	Thomsen et al. (2004)
PAOmix	Most *Accumulibacter* sp.	PAO462 (CCGTCATCTACWCAGGGTATTAAC), PAO651 (CCCTCTGCCAAACTCCAG), PAO846 (GTTAGCTACGGCACTAAAAGG)	35/80	Crocetti et al. (2000)
AZA645	*Azoarcus* cluster	GCCGTACTCTAGCCGTGC	20/225	Hess et al. (1997)
Thau646	*Thauera* sp.	TCTGCCGTACTCTAGCCTT	45/40	Lajoie et al. (2000)
ACI208	*Acidovorax* spp.	CGCGCAAGGCCTTGC	20/225	Amann et al. (1996)
ZRA23a	Most members of the *Zooglea* lineage, not *Z. resiniphila*;	CTGCCGTACTCTAGTTAT	35/80	Rosselló-Mora et al. (1995)
AT1458	*Azoarcus*–*Thauera* cluster	GAATCTCACCGTGGTAAGCGC	50/28	Rabus et al. (1999)
BET65	Bacteria closely related to *Comamonadaceae*	CAGTTGCCCCGCGTACCG	30/112	Kong et al. (2007)
BET135	Bacteria closely related to *Rhodocyclaceae* clones	ACGTTATCCCCCACTCAATGG	45/40	Kong et al. (2007)
Nos174	Cultured *Tetrasphaera* PAO	GCTCCGTCTCGTATCCGG	30/112	Nguyen et al. (2011)

### Data analysis

The data analysis was performed using STATISTICA 10.0 (StatSoft). For the analyses, the data from a period of a stable reactor operation at a given temperature were taken (ammonium concentration in the effluent was close to 0). A value of *p* ≤ 0.05 was defined as significant. The distribution was analyzed by the Shapiro–Wilk test, and homogeneity of variance by the Levene’s test. The studies used ANOVA and RIR Tukey’s test and Pearson correlation coefficient.

## Results

### Biomass characteristics, DO profiles

The concentrations of biomass in the experimental stages were about 15,500 mg TSS/L at 29 °C, 15,000 mg TSS/L at 25 °C, and 12,000 mg TSS/L at 20 °C. The concentration of total suspended solids in the effluent varied between 500 and 600 mg TSS/L. The sludge volumetric index (SVI) in stage 1 and 2 averaged 7.94 ± 0.3 mL/g TSS and 7.97 ± 0.2 mL/g TSS, respectively. At the lowest applied temperature, SVI value increased to 11.69 ± 0.4 mL/g TSS. Presuming that biomass particles with diameters >0.125 mm are granules, granules constituted 77, 80 and 83 % of biomass at 29, 25 and 20 °C, respectively. Independent of the temperature, granules with diameters in the range from 0.125 to 0.500 mm predominated. The time-courses of dissolved oxygen showed that, in all stages, DO concentration declined to about 3 mg/L during reactor feeding with a fresh portion of wastewater and remained at this level for the next 3 h of the cycle. After 4–5 h of the cycle, ammonia and easily biodegradable COD were oxidized, therefore DO increased to 7–8 mg/L.

### Carbon removal by aerobic granules

**Fig. 1:**
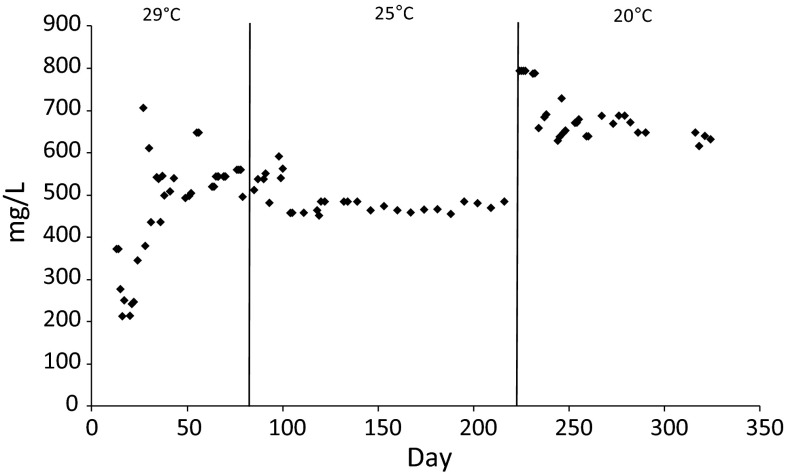
Concentration of COD in the GSBR effluent depending of the operational temperature

The average concentration of organic compounds fed to the reactor, expressed as a chemical oxygen demand (COD), during operation of the reactor was 972.6 ± 133.1 mg/L. The average concentrations of COD in the treated effluents was 551.5 ± 42.0, 487.4 ± 35.6, and 657.6 ± 21.8 mg/L in stage 1, 2 and 3, respectively (Fig. 1). The organic content in the effluent from the GSBR operated at 25 °C was significantly lower that at the other two temperatures (*p* < 0.000 and *p* < 0.000 for 29 and 20 °C, respectively). The COD concentration in the effluent from the GSBR operated at 29 °C was significantly lower than in the effluent from GSBR operated at 20 °C (*p* < 0.000). The COD removal efficiency at 25 °C (27.3 ± 2.5 %) was significantly higher than at 29 °C (18.8 ± 3.6 %, *p* < 0.000), and 20 °C (21.19 ± 1.3 %, *p* < 0.000).

**Fig. 2:**
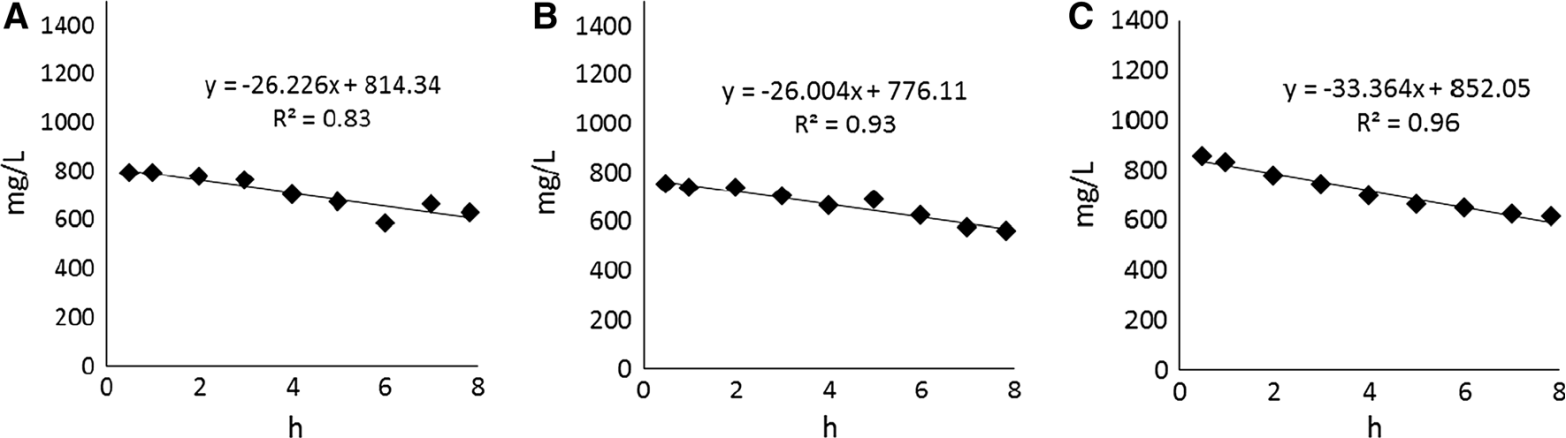
Changes of COD concentrations in the GSBR cycle at **a** 29 °C, **b** 25 °C and **c** 20 °C

In the period of stable reactor operation at a given temperature, changes of COD concentration were measured during the operating cycle of the GSBR. The COD decreased linearly in the cycle and COD removal rates varied from 26.0 mg/(L h) at 25 °C to 33.38 mg/(L h) at 20 °C (Fig. 2).

### Nitrogen removal by aerobic granules

The diluted landfill leachate supplied to the experimental GSBR had an average concentration of ammonium nitrogen of 303.8 ± 23.1 mg/L. In the first, second and third stage of the study, ammonium nitrogen accounted for about 70, 66 and 69 % of the total Kjeldahl nitrogen (TKN) in the influent, respectively.

**Fig. 3:**
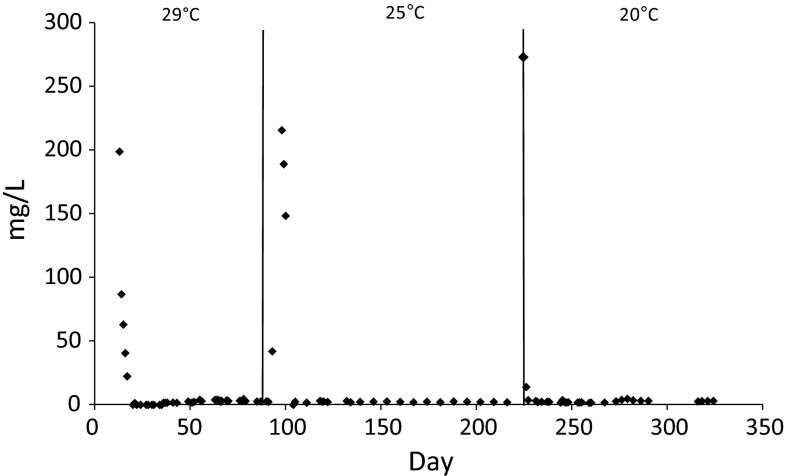
Concentration of ammonium nitrogen in the GSBR effluent depending of the operational temperature

Measurement of ammonium nitrogen in the effluent from the reactor in stage 1 (operating temperature 29 °C) commenced from cycle 18 and it was 235.2 mg/L (Fig. 3). From cycle 60 of the GSBR operation, ammonium nitrogen concentration in the effluent was close to 0 mg/L. Lowering the process temperature to 25 °C resulted in the collapse of the oxidation of ammonium nitrogen; its concentration in the effluent increased to 215.6 mg/L. The biomass adapted quickly and starting from cycle 321 ammonium nitrogen in the leachate was again fully oxidized. Lowering the operating temperature to 20 °C resulted in an increase in the concentration of ammonium nitrogen in the effluent to about 270 mg/L in cycles 672–675. From cycle 681 complete removal of ammonium nitrogen in the reactor was again observed. The concentration of ammonium nitrogen in the effluent was significantly lower (*p* < 0.003) in the reactor operated at 25 °C than in the reactor operated at 29 °C.

**Fig. 4:**
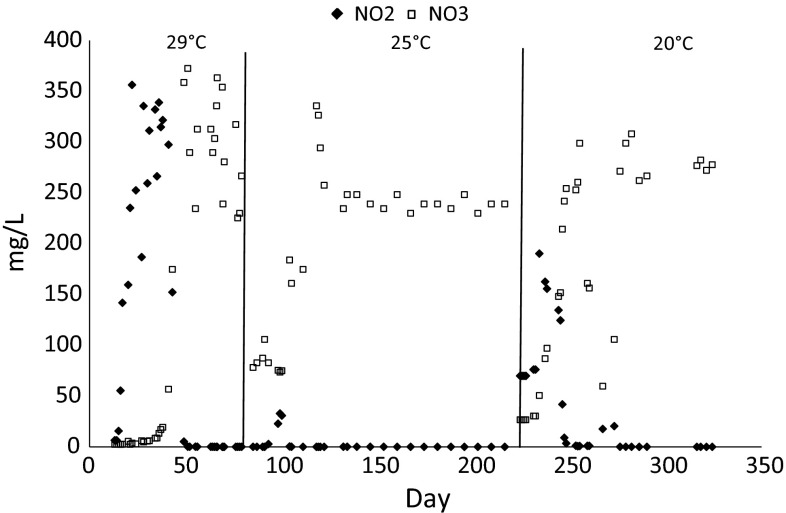
Concentration of nitrites and nitrates in the GSBR effluent depending of the operational temperature

In the reactor operated at 29 °C, the concentration of nitrites in the effluent between cycles 42 and 150 was high with a maximum value of 356.8 mg/L in cycle 66. From cycle 150 of the reactor operation, concentration of nitrites was close to 0 mg/L. Lowering the operational temperature to 25 °C increased concentration of nitrites to about 30 mg/L in cycles 279–300. Further lowering of the temperature in stage 3 increased the concentration of nitrites up to 190.3 mg/L in cycle 702. From cycle 762, concentration of nitrites in the treated leachate remained below 1 mg/L (Fig. 4).

From cycle 100, complete nitrification (nitrification to nitrate as a final product) was observed in the reactor, and the concentration of nitrates in the effluent averaged 300 mg/L (Fig. 4). After changing the operational temperatures of the reactor to 25 °C and then to 20 °C a sudden drop in concentration of nitrates was observed. From about cycles 400 and 780, nitrate concentration in the outflow stabilized at a level of 250 and 267 mg/L, respectively. TKN concentrations in the effluent from the GSBR at the end of each experimental stage averaged 37.4 ± 2.1, 43.2 ± 3.3 and 32.2 ± 1.9 mg/L respectively at the operating temperatures of 29, 25 and 20 °C.

**Fig. 5:**
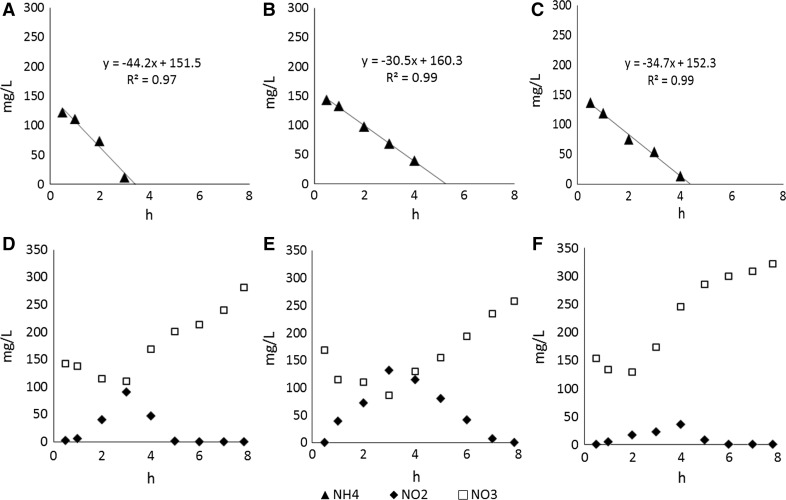
Changes of ammonium nitrogen, nitrite and nitrate concentrations in the GSBR cycle at **a** 29 °C, **b** 25 °C and **c** 20 °C

The highest rate of ammonium nitrogen removal in the GSBR cycle was at 29 °C [44.2 mg/(L h)], while at two others operational temperatures it was about 30 mg/(L h). In the reactor operated at 29 °C, ammonium was fully removed from the leachate after about 3 h of the reactor cycle, while at 25 and 20 °C after more than 4 h (Fig. 5). Examination of changes in the concentration of oxidized forms of nitrogen in the first stage, showed the increase in the concentration of nitrites to about 90 mg/L in the third hour of the cycle. From about the sixth hour of the cycle, nitrites concentration was close to zero. In the reactor operated at 25 °C, nitrite concentration peaked in the third hour of the cycle. The lowest nitrite accumulation was observed in the reactor operated at 20 °C when, in the 4th hour of the cycle, nitrites reached 35.6 mg/L (Fig. 5). At all operational temperatures, the concentration of nitrates decreased in the first hours of the cycle. Then it increased steadily until the end of the cycle up to about 280, 260 and 320 mg/L in the GSBR operated at 29, 25 and 20 °C, respectively (Fig. 5).

The overall efficiency of nitrogen compounds transformation in the experimental stages was calculated taking into account the results from a stable reactor operation at a given temperature. Nitrification efficiency was 80.9 ± 5.5 %, 81.3 ± 6.6 % and 81.4 ± 3.16 % at 29, 25 and 20 °C, respectively. The differences in the effectiveness of nitrification were not significant. At 29 °C, the efficiency of denitrification averaged 10.6 ± 6.2 %. Lowering operational temperature to 25 and 20 °C significantly increased the efficiency of denitrification in comparison with 29 °C (*p* = 0.011 and *p* = 0.026 at 25 and 20 °C, respectively). The denitrification efficiency in the reactor operated at 25 °C averaged 25.5 ± 10.8 %, while in the reactor operated at 20 °C it was 21.4 ± 15.0 %. The average efficiency of total nitrogen removal equaled 36.8 ± 10.9 % at 25 °C, 32.1 ± 14.0 % at 20 °C and 19.2 ± 10.8 % at 29 °C. The average total nitrogen removal efficiency was significantly higher (*p* = 0.002) at 25 °C, and 20 °C (*p* = 0.009) compared to 29 °C.

### FISH

Fluorescence in situ hybridization was carried out to investigate how the microbial structure of granules changed with lowering the operational temperature of the GSBR treating landfill leachate (Table 2). The biovolume of all bacteria was about 50–60 % in all slides. Ammonium oxidation in aerobic granules was carried out by both aerobic and anaerobic ammonium-oxidizing bacteria. The abundance of AOB was the highest in the biomass from the GSBR operated at 25 °C and reached 16.6 ± 11.9 %. Anammox bacteria were less abundant and their percentage varied from 2.5 ± 0.9 % at 25 °C to 4.2 ± 1.9 % at 20 °C. Bacteria from *Accumulibacter* sp., *Thauera* sp., cultured *Tetrasphaera* PAO and *Azoarcus*–*Thauera* cluster were present in the granules independent of the operational temperature, with an abundance mostly at a level of a few percent. The analysis indicated, however, bacterial groups that were favored by lowering the operational temperature of the process. These were members of the *Zooglealineage* (not *Z. resiniphila*), bacteria closely related to *Comamonadaceae*, *Curvibacter* sp., *Azoarcus* cluster, *Rhodobacter*, *Roseobacter* and *Acidovorax* spp. These groups of microorganisms were absent at the highest operational temperatures and their abundance gradually increased with lowering of temperature. Bacteria of the genus *Paracoccus*, on the other hand, diminished with the decreasing of the temperature of the GSBR operation.

**Table 2:** Percentage abundance of nitrogen-converting microorganisms in aerobic granules depending on the operational temperature of the treatment

Target	29 °C	25 °C	20 °C
Betaproteobacterial ammonium-oxidizing bacteria	9.3 ± 5.9	16.6 ± 11.9	12.3 ± 9.7
All Anammox bacteria	3.3 ± 1.9	2.5 ± 0.9	4.2 ± 1.9
Most *Accumulibacter* sp.	3.6 ± 1.6	3.3 ± 1.2	7.8 ± 7.8
*Thauera* sp.	3.9 ± 3.6	2.4 ± 0.4	4.3 ± 3.8
Most members of the *Zooglea* *lineage*. not *Z. resiniphila*;	0.0	4.1 ± 2.8	8.2 ± 5.0
Bacteria closely related to *Comamonadaceae*	0.0	5.6 ± 3.5	10.0 ± 7.1
Bacteria closely related to *Rhodocyclaceae* clones	0.0	0.0	0.0
Cultured *Tetrasphaera* PAO	6.4 ± 4.2	4.3 ± 2.1	10.7 ± 8.4
*Curvibacter* sp.	0.0	4.8 ± 1.6	6.4 ± 2.4
*Azoarcus* cluster	0.0	2.8 ± 0.6	9.2 ± 6.9
*Paracoccus* sp.	4.6 ± 3.1	4.2 ± 2.9	0.0
*Rhodobacter* sp., *Roseobacter* sp.	0.0	0.0	1.5 ± 1.2
*Azoarcus*–*Thauera* cluster	3.0 ± 1.2	2.7 ± 0.2	4.9 ± 1.7
*Acidovorax* spp.	0.0	5.4 ± 3.6	6.0 ± 5.7

A correlation matrix was constructed to relate the wastewater composition and treatment efficiency of the process with the abundance of particular groups of bacteria in aerobic granules. Higher organics load supported the higher presence of *Azoarcus* sp. Abundances of cultured *Tetrasphaera* PAO and Anammox bacteria were correlated with the COD concentration in the effluent. Nitrites concentration in the effluent was positively correlated with most *Accumulibacter* (PAO mix polyphosphate-accumulating microorganisms) and *Rhodobacter* sp. and *Roseobacter* sp. *Acidovorax* spp. correlated negatively with concentration of nitrates in the effluent and positively with the efficiency of nitrification.

## Discussion

The results of this study indicate that gradual acclimatization of aerobic granular biomass to ambient temperature (20 °C) maintains the effectiveness of ammonium oxidation in leachate and improves the overall effectiveness of N removal. These results also show which groups of bacteria in aerobic granules are responsible for nitrogen conversions at different temperatures during the treatment of high nitrogen wastewater.

In our study, independent of the temperature, the COD removal efficiency did not exceed 50 %. This low COD removal efficiency may be explained by the presence of hard-to-biodegrade compounds in the leachate fed to the reactor. The low biodegrability of leachate resulted in a slow linear decrease of COD concentration in the GSBR cycle. In contrast, Wang et al. (2009) reported that during treatment of wastewater with easily biodegradable organics (COD = 723 mg/L, N-NH_4_^+^ = 71 mg/L), a significant decrease in COD concentration of about 60 % was observed in the anoxic phase at the beginning of the cycle, and then a slow decrease in COD concentration occurred during the rest of the cycle.

Despite a very low amount of easily biodegradable COD, the unique settling properties of aerobic granules, shown by very low sludge volume index values, enabled good accumulation of biomass in the reactor. The average concentrations of biomass in the GSBR operated at 29 and at 25 °C were over 15 g TSS/L, whereas biomass concentration at 20 °C dropped to 12 g TSS/L. The decrease in biomass concentration in the GSBR with the lowering of the operation temperature can be explained by the fact that more efficient denitrification at lower temperatures caused organics to be removed for the reduction of oxidized forms of nitrogen, so less organics were available for biomass growth. The observed biomass concentrations are similar to those in other studies in aerobic granular sludge systems (Yilmaz et al. 2008; Liu et al. 2009) but significantly higher than those in activated sludge systems, where the biomass concentration varies between 2 and 4 g TSS/L. These high concentrations of biomass in GSBRs are desirable because they allow the use of smaller reactors than in systems with conventional activated sludge. The concentration of biomass in the outflow from the GSBR in all three stages was higher than 400 mg TSS/L, so a secondary clarifier or membrane module should be included in the treatment line for leachate purification. In general, such high concentrations of TSS in the effluent from GSBR are caused by the very short settling that is necessary to maintain the pressure that is necessary for biomass to granulate.

Higher temperatures resulted in higher ammonium removal rates. Nitrites were present in the GSBR cycle and their accumulation diminished when the operational temperature was lowered. Their concentrations at the end of the cycle were close to zero and indicated effective complete nitrification and the reduction of oxidized forms of nitrogen. Higher concentrations of nitrite in the effluent were only observed just after changing the operational temperatures—this phenomena is often observed in the start-up stage of aerobic granular sludge reactors and at a low COD/N ratio in wastewater (Bao et al. 2009). The present study showed that ammonium oxidation was efficiently carried out by both aerobic and anaerobic ammonium-oxidizing bacteria that coexisted in the multi-layer structure of the granules despite constant aeration of the reactor. Lowering the temperature of the process caused only short-term disturbances in ammonium oxidation. This can be explained by protection of nitrifying bacteria from the inhibitory effect of free ammonium at higher temperatures by extracellular polymeric substances in the structure of aerobic granules. Anammox bacteria abundance positively correlated with COD in the effluent, which may confirm that they use organics for their growth (Strous et al. 2006; Winkler et al. 2012). The presence of Anammox bacteria in the system is favorable during the treatment of hard-to-biodegrade wastewater with high ammonium load, such as landfill leachate, because at least some part of the nitrogen can be removed without the use of organic carbon sources.

The difference in efficiency of the nitrogen conversion underlines significantly better nitrogen removal conditions using aerobic granular sludge at 20 and 25 °C than at 29 °C. The molecular results shed light on dynamics of microbial community in aerobic granules under high nitrogen loading and variable temperature. Our study showed that under conditions of low availability of easily biodegradable organics and high nitrogen load, biomass was dominated by microorganisms involved in the metabolism of nitrogen compounds. Denitrification was carried out by many different groups of bacteria and the study allowed identification of temperature-insensitive and temperature-sensitive denitrifiers. The first group included bacteria from *Accumulibacter* sp., *Thauera* sp. cultured *Tetrasphaera* PAO and *Azoarcus*–*Thauera* cluster. The above-mentioned groups of bacteria were observed throughout the whole experiment, suggesting their resistance to high nitrogen loading and low COD availability. All these groups of bacteria have been identified as dominant denitrifying populations in activated sludge from biological nutrient removal plants (Kong et al. 2007; Thomsen et al. 2007; Nielsen et al. 2010). The members of the genus *Tetrasphaera* exhibit the characteristics of polyphosphate-accumulating organisms (PAOs), and contain functional genes for denitrification (Carvalho et al. 2007; Kristiansen et al. 2013). The presence of denitrifying PAOs in the treatment system is advantageous because they reduce the amount of COD required to support both denitrification and phosphorus removal (van Loosdrecht et al. 1998). Bassin et al. (2012) explained the better phosphate removal efficiency in the reactor operated at 20 °C than in the reactor operated at 30 °C by the fact that a considerable fraction of the phosphate removal was coupled to more efficient denitrification (denitrifying dephosphatation).

Lowering the temperature of the GSBR operation induced growth of members of the *Zooglealineage* (not *Z. resiniphila*), bacteria closely related to *Comamonadaceae*, *Curvibacter* sp., *Azoarcus* cluster, *Rhodobacter* sp., *Roseobacter* sp. and *Acidovorax* spp. in aerobic granules. These groups of bacteria have been identified in activated sludge wastewater treatment plants with nitrogen removal and in laboratory-scale installations with aerobic granules (e.g. Kong et al. 2007; Thomsen et al. 2007; Gonzalez-Gil and Holliger 2011). The members of the *Comamonadaceae* family may play a major role in denitrification in aerobic granular sludge systems supplied with acetate as an external carbon source (Ginige et al. 2005, Adav et al. 2010). The above mentioned groups of microorganisms can utilize different substrates e.g. *Curvibacter*-related bacteria take up only mixed amino acids, whereas *Azoarcus* utilize short-chain fatty acids and aminoacids. This enables their simultaneous occurrence in the biomass (Thomsen et al. 2007). Most *Curvibacter*-related bacteria are able to utilize both nitrate and nitrite as an electron acceptor under both aerobic and anoxic conditions (Thomsen et al. 2004). This dictates the versatility of these bacteria. Denitrifiers can grow in a wide range of temperatures and 20 °C is most often regarded as the optimum. However, the activity of denitrification enzymes decreases with decreasing temperature (Saleh-Lakha et al. 2009). Our study showed that the increase in the number of the denitrifiers mentioned above with decreasing temperature compensated for the decrease in enzymatic activity; due to their higher number, the microorganisms were able to denitrify at 20 °C with a net efficiency similar to that observed at 25 °C (no significant differences) and significantly better than at 29 °C.

In our study, cultured *Tetrasphaera* PAO abundance positively correlated with COD concentration in the effluent. It is possible that the increase in COD in the effluent was caused by the production of soluble polymeric substances by *Thauera* sp. (Pollock et al. 1998; Allen et al. 2004). A positive correlation observed between *Accumulibacter* (PAO mix polyphosphate-accumulating microorganisms) presence and nitrites concentration in the effluent can be explained by a very high ability of *Accumulibacter* sp. to use nitrites as an electron acceptor during CO_2_ assimilation in the presence of the complex organic substrate mixture (Morgan-Sagastume et al. 2008). On the other hand, a negative correlation between *Acidovorax* spp. abundance and nitrates concentration in the effluent suggests that these bacteria are heavily involved in denitrification from nitrates. The presence of *Acidovorax* spp. is favorable for nitrogen removal since these genera perform complete nitrate reduction to N_2_ (Gentile et al. 2007). Previous study has shown that *Paracoccus* sp. dominated in the biomass from reactors with aerobic granules operated at higher temperatures (26–27 °C) under high nitrogen loading and with an anoxic phase at the beginning of the cycle (Cydzik-Kwiatkowska 2015). This study showed however, that they are sensitive to temperature changes and disappeared from the biomass treating leachate at ambient temperatures.

## Conclusions

Efficient removal of nitrogen at ambient temperatures translates into lower operating costs for a sewage treatment system. In the present study, it was shown that gradual adaptation of the biomass to lowering of operational temperatures enabled full ammonium oxidation from leachate by aerobic granules at room temperature and significantly improved denitrification efficiency. Granules comprised 77–83 % of the biomass, and granules with diameters in the range from 0.125 to 0.500 mm predominated. Ammonium oxidation was carried out by both aerobic and anaerobic ammonium-oxidizing bacteria. Operation of the reactor at ambient temperatures decreased nitrite accumulation in the GSBR cycle and favored the growth of complete denitrifiers in the biomass, which should diminish the nitrous oxide emissions from the system. *Accumulibacter* sp., *Thauera* sp. cultured *Tetrasphaera* PAO and *Azoarcus*–*Thauera* cluster were present in granules at high nitrogen loading at all investigated temperatures, while the abundance of denitrifiers of *Zooglealineage* (not *Z. resiniphila*), bacteria closely related to *Comamonadaceae*, *Curvibacter* sp., *Azoarcus* cluster, *Rhodobacter* sp., *Roseobacter* sp. and *Acidovorax* spp. in the biomass increased with decreasing temperature.

## References

[CR1] Adav SS, Lee DJ, Lai JY (2010). Microbial community of acetate utilizing denitrifiers in aerobic granules. Appl Microbiol Biotechnol.

[CR2] Allen MS, Welch KT, Prebyl BS, Baker DC, Meyers AJ, Sayler GS (2004). Analysis and glycosyl composition of the exopolysaccharide isolated from the floc-forming wastewater bacterium *Thauera* sp. MZ1T. Environ Microbiol.

[CR3] Amann RI, Binder BJ, Olson RJ, Chisholm SW, Devereux R, Stahl DA (1990). Combination of 16S rRNA-targeted oligonucleotide probes with flow cytometry for analyzing mixed microbial populations. Appl Environ Microbiol.

[CR4] Amann R, Ludwig W, Schulze R, Spring S, Moore E, Schleifer KH (1996). rRNA-targeted oligonucleotide probes for the identification of genuine and former pseudomonads. Syst Appl Microbiol.

[CR5] APHA (1998). Standard methods for the examination of water and wastewater.

[CR6] Bao R, Yu S, Shi W, Zhang X, Wang Y (2009). Aerobic granules formation and nutrients removal characteristics in sequencing batch airlift reactor (SBAR) at low temperature. J Hazard Mater.

[CR7] Bassin JP, Pronk M, Kraan R, Kleerebezem R, van Loosdrecht MCM (2011). Ammonium adsorption in aerobic granular sludge, activated sludge and anammox granules. Water Res.

[CR8] Bassin JP, Kleerebezem R, Dezotti M, van Loosdrecht MCM (2012). Simultaneous nitrogen and phosphate removal in aerobic granular sludge reactors operated at different temperatures. Water Res.

[CR9] Carvalho G, Lemos PC, Oehmen A, Reis MAM (2007). Denitrifying phosphorus removal: linking the process performance with the microbial community structure. Water Res.

[CR10] Crocetti GR, Hugenholtz P, Bond PL, Schuler A, Keller J, Jenkins D, Blackall LL (2000). Identification of polyphosphate-accumulating organisms and design of 16S rRNA-directed probes for their detection and quantitation. Appl Environ Microbiol.

[CR11] Cydzik-Kwiatkowska A (2015). Bacterial structure of aerobic granules is determined by aeration mode and nitrogen load in the reactor cycle. Bioresour Technol.

[CR12] Cydzik-Kwiatkowska A, Zielińska M, Bernat K, Wojnowska-Baryła I, Truchan T (2013). Treatment of high-ammonium anaerobic digester supernatant by aerobic granular sludge and ultrafiltration processes. Chemosphere.

[CR13] Daims H, Bruhl A, Amann R, Schleifer KH, Wagner M (1999). The domain-specific probe EUB338 is insufficient for the detection of all Bacteria: development and evaluation of a more comprehensive probe set. Syst Appl Microbiol.

[CR14] de Kreuk MK, van Loosdrecht MCM (2004). Selection of slow growing organisms as a means for improving aerobic granular sludge stability. Water Sci Technol.

[CR15] de Kreuk MK, van Loosdrecht MCM (2006). Formation of aerobic granules with domestic sewage. J Environ Eng.

[CR16] de Kreuk MK, Pronk M, van Loosdrecht MCM (2005). Formation of aerobic granules and conversion processes in an aerobic granular sludge reactor at moderate and low temperatures. Water Res.

[CR17] Gao D, Liu L, Liang H, Wu W-M (2011). Aerobic granular sludge: characterization, mechanism of granulation and application to wastewater treatment. Crit Rev Biotechnol.

[CR18] Gentile ME, Jessup CS, Nyman JL, Criddle CS (2007). Correlation of functional instability and community dynamics in denitrifying dispersed-growth reactors. Appl Environ Microbiol.

[CR19] Ginige MP, Keller J, Blackall LL (2005). Investigation of an acetate-fed denitrifying microbial community by stable-isotope probing, full-cycle rRNA analysis, and fluorescence in situ hybridization-microautoradiography. Appl Environ Microbiol.

[CR20] Giuliano L, De Domenico M, De Domenico E, Hofle MG, Yakimov MM (1999). Identification of culturable oligotrophic bacteria within naturally occurring bacterioplankton communities of the Ligurian sea by 16S rRNA sequencing and probing. Microbial Ecol.

[CR21] Gonzalez-Gil G, Holliger C (2011). Dynamics of microbial community structure of and enhanced biological phosphorus removal by aerobic granules cultivated on propionate or acetate. Appl Environ Microbiol.

[CR22] Halim MHA, Anuar AN, Azmi SI, Jamal NSA, Wahab NA, Ujang Z, Shraim A, Bob MM (2015). Aerobic sludge granulation at high temperatures for domestic wastewater treatment. Bioresour Technol.

[CR23] Hess A, Zarda B, Hahn D, Haner A, Stax D, Hohener P, Zeyer J (1997). In situ analysis of denitrifying toluene- and m-xylene-degrading bacteria in a diesel fuel-contaminated laboratory aquifer column. Appl Environ Microbiol.

[CR24] Kishida N, Tsuneda S, Kim JH, Sudo R (2009). Simultaneous nitrogen and phosphorus removal from high-strength industrial wastewater using aerobic granular sludge. J Environ Eng.

[CR25] Kong Y, Xia Y, Nielsen JL, Nielsen PH (2007). Structure and function of the microbial community in a full-scale enhanced biological phosphorus removal plant. Microbiology.

[CR26] Kristiansen R, Nguyen HT, Saunders AM, Nielsen JL, Wimmer R, Le VQ, McIlroy SJ, Petrovski S, Seviour RJ, Calteau A, Nielsen KL, Nielsen PH (2013). A metabolic model for members of the genus *Tetrasphaera* involved in enhanced biological phosphorus removal. ISME J.

[CR27] Kulikowska D (2009). Charakterystyka oraz metody usuwania zanieczyszczeń organicznych z odcieków pochodzących z ustabilizowanych składowisk odpadów komunalnych. Ecol Chem Eng.

[CR28] Lajoie CA, Dayton AC, Gregory IR, Sayler GS, Taylor DE, Meyers AJ (2000). Zoogleal clusters and sludge dewatering potential in an industrial activated-sludge wastewater treatment plant. Water Environ Res.

[CR29] Liu XW, Sheng GP, Yu HQ (2009). Physicochemical characteristics of microbial granules. Biotechnol Adv.

[CR30] Mobarry BK, Wagner M, Urbain V, Rittmann BE, Stahl DA (1996). Phylogenetic probes for analyzing abundance and spatial organization of nitrifying bacteria. Appl Environ Microbiol.

[CR31] Morgan-Sagastume F, Nielsen JL, Nielsen PH (2008). Substrate-dependent denitrification of abundant probe-defined denitrifying bacteria in activated sludge. FEMS Microbiol Ecol.

[CR32] Mosquera-Corral A, de Kreuk MK, Heijnen JJ, van Loosdrecht MCM (2005). Effects of oxygen concentration on N-removal in an aerobic granular sludge reactor. Water Res.

[CR33] Neef A, Zaglauer A, Meier H, Amann R, Lemmer H, Schleifer KH (1996). Population analysis in a denitrifying sand filter: conventional and in situ identification of *Paracoccus* spp. in methanol-fed biofilms. Appl Environ Microbiol.

[CR34] Nguyen HTT, Le VQ, Hansen AA, Nielsen JL, Nielsen PH (2011). High diversity and abundance of putative polyphosphate-accumulating *Tetrasphaera*-related bacteria in activated sludge systems. FEMS Microbiol Ecol.

[CR35] Nielsen PH, Nguyen HTT, McIlroy S, Mielczarek AT, Seviour R, Nielsen PH, Daims H, Lemmer H (2009). Identification of polyphosphate-accumulating and glycogen-accumulating organisms by FISH. FISH Handbook for biological wastewater treatment: identification and quantification of microorganisms in activated sludge and biofilms by FISH.

[CR36] Nielsen PH, Mielczarek AT, Kragelund C, Nielsen JL, Saunders AM, Kong Y, Hansen AA, Vollertsen J (2010). A conceptual ecosystem model of microbial communities in enhanced biological phosphorus removal plants. Water Res.

[CR100] Pollock TJ, van Workum WAT, Thorne L, Mikolajczak MJ, Yamazaki M, Kijne JW, Armentrout RW (1998). Assignment of biochemical functions to glycosyl transferase genes which are essential for biosynthesis of exopolysaccharides in *Sphingomonas* strain S88 and *Rhizobium leguminosarum*. J Bacteriol.

[CR37] Rabus R, Wilkes H, Schramm A, Harms G, Behrends A, Amann R, Widdel F (1999). Anaerobic utilization of alkylbenzenes and n-alkanes from crude oil in an enrichment culture of denitrifying bacteria affiliating with the beta-subclass of Proteobacteria. Environ Microbiol.

[CR38] Rosselló-Mora RA, Wagner M, Amann R, Schleifer KH (1995). The abundance of *Zoogloearamigera* in sewage treatment plants. Appl Environ Microbiol.

[CR39] Saleh-Lakha S, Shannon KE, Henderson SL, Goyer C, Trevors JT, Zebarth BJ, Burton DL (2009). Effect of pH and temperature on denitrification gene expression and activity in *Pseudomonas mandelii*. Appl Environ Microbiol.

[CR40] Schmid M, Walsh K, Webb R, Rijpstra WIC, van de Pas-Schoonen K, Verbruggen MJ, Hill T, Moffett B, Fuerst J, Schouten S, Damsté JSS, Harris J, Shaw P, Jetten M, Strous M (2003). Two new species of anaerobic ammonium oxidizing bacteria. Syst Appl Microbiol.

[CR41] Song Z, Ren N, Zhang K, Tong L (2009). Influence of temperature on the characteristics of aerobic granulation in sequencing batch airlift reactors. J Environ Sci.

[CR42] Strous M, Pelletier E, Mangenot S (2006). Deciphering the evolution and metabolism of an anammox bacterium from a community genome. Nature.

[CR43] Thomsen TR, Nielsen JL, Ramsing NB, Nielsen PH (2004). Micromanipulation and further identification of FISH-labelled microcolonies of a dominant denitrifying bacterium in activated sludge. Environ Microbiol.

[CR44] Thomsen TR, Kong Y, Nielsen PH (2007). Ecophysiology of abundant denitrifying bacteria in activated sludge. FEMS Microbiol Ecol.

[CR45] van Loosdrecht MCM, Brandse FA, Vries AC (1998). Upgrading of wastewater treatment processes for integrated nutrient removal—the BCFS process. Water Sci Technol.

[CR46] Wang F, Lu S, Wei Y, Ji M (2009). Characteristics of aerobic granule and nitrogen and phosphorus removal in a SBR. J Hazard Mater.

[CR47] Wang X, Jia M, Chen X, Xu Y, Lin X, Kao CM, Chen S (2014). Greenhouse gas emissions from landfill leachate treatment plants: a comparison of young and aged landfill. Waste Manag.

[CR48] Wei Y, Ji M, Li R, Qin F (2012). Organic and nitrogen removal from landfill leachate in aerobic granular sludge sequencing batch reactors. Waste Manag.

[CR49] Winkler MK, Bassin JP, Kleerebezem R, de Bruin LM, van den Brand TP, van Loosdrecht MCM (2011). Selective sludge removal in a segregated aerobic granular biomass system as a strategy to control PAO–GAO competition at high temperatures. Water Res.

[CR50] Winkler MK, Yang J, Kleerebezem R, Plaza E, Trela J, Hultman B, van Loosdrecht MCM (2012). Nitrate reduction by organotrophic Anammox bacteria in a nitritation/anammox granular sludge and a moving bed biofilm reactor. Bioresour Technol.

[CR51] Wu JJ, Wu C-C, Ma H-W, Chang C-C (2004). Treatment of landfill leachate by ozone-based advanced oxidation processes. Chemosphere.

[CR52] Yilmaz G, Lemaire R, Keller J, Yuan Z (2008). Simultaneous nitrification, denitrification, and phosphorus removal from nutrient-rich industrial wastewater using granular sludge. Biotechnol Bioeng.

[CR53] Yu X, Wan C, Lei Z, Liu X, Zhang Y, Lee D-J, Tay J-H (2014). Adsorption of ammonium by aerobic granules under high ammonium levels. J Taiwan Inst Chem Eng.

